# The core outcome measures in effectiveness trials (COMET) initiative: five years on

**DOI:** 10.1186/1745-6215-16-S2-P69

**Published:** 2015-11-16

**Authors:** Paula Williamson, Douglas Altman, Jane Blazeby, Michael Clarke, Elizabeth Gargon, Sarah Gorst, Sean Tunis

**Affiliations:** 1 University of Liverpool, Liverpool, UK; 2 University of Oxford, Oxford, UK; 3 University of Bristol, Bristol, UK; 4 Queen's University Belfast, Belfast, UK; 5 Center for Medical Technology and Policy, Baltimore, MD, USA

## 

Core outcome sets (COS) can reduce waste in research, through measurement of an agreed set of outcomes across all trials in a particular area of health. The COMET Initiative was launched in 2010 to (i) raise awareness of problems with outcomes in trials; (ii) encourage COS development and uptake; (iii) promote patient and public involvement in COS development; (iv) provide resources to facilitate this; and (v) encourage evidence-based COS development.

This talk will review progress and challenges over our first five years. It will describe our work with multiple stakeholders to facilitate engagement, including funders, trialists, patient organisations, systematic reviewers, editors, industry, regulators, and guideline developers. The perception that COMET was a ‘UK thing’ has been dispelled, with the establishment of an International Advisory Group and meetings in Rome (2014) and Calgary (2015).

Highlights include the development of a searchable repository of COS studies, and completion of the first comprehensive systematic review of published COS (recently updated to December 2014). Over 7800 searches have been done of the online repository, most visitors in 2014 were from outside the UK and we will present findings from a pop-up survey of why people search it.

Promoting broader uptake of COS by researchers and securing funding for initiatives aimed at reducing waste in research remain major challenges. As awareness of the need for COS continues to grow and knowledge of the COMET Initiative increases, this is an appropriate moment to present our future proposals, including a research agenda, to the trials methodology community.

